# Effects of Mobile Augmented Reality Learning Compared to Textbook Learning on Medical Students: Randomized Controlled Pilot Study

**DOI:** 10.2196/jmir.2497

**Published:** 2013-08-20

**Authors:** Urs-Vito Albrecht, Kristian Folta-Schoofs, Marianne Behrends, Ute von Jan

**Affiliations:** ^1^PL Reichertz Institute for Medical InformaticsHannover Medical SchoolHannoverGermany; ^2^Institute of PsychologyUniversity of HildesheimHildesheimGermany

**Keywords:** problem-based learning, cellular phone, education, medical, emotions

## Abstract

**Background:**

By adding new levels of experience, mobile Augmented Reality (mAR) can significantly increase the attractiveness of mobile learning applications in medical education.

**Objective:**

To compare the impact of the heightened realism of a self-developed mAR blended learning environment (mARble) on learners to textbook material, especially for ethically sensitive subjects such as forensic medicine, while taking into account basic psychological aspects (usability and higher level of emotional involvement) as well as learning outcomes (increased learning efficiency).

**Methods:**

A prestudy was conducted based on a convenience sample of 10 third-year medical students. The initial emotional status was captured using the “Profile of Mood States” questionnaire (POMS, German variation); previous knowledge about forensic medicine was determined using a 10-item single-choice (SC) test. During the 30-minute learning period, the students were randomized into two groups: the first group consisted of pairs of students, each equipped with one iPhone with a preinstalled copy of mARble, while the second group was provided with textbook material. Subsequently, both groups were asked to once again complete the POMS questionnaire and SC test to measure changes in emotional state and knowledge gain. Usability as well as pragmatic and hedonic qualities of the learning material was captured using AttrakDiff2 questionnaires. Data evaluation was conducted anonymously. Descriptive statistics for the score in total and the subgroups were calculated before and after the intervention. The scores of both groups were tested against each other using paired and unpaired signed-rank tests. An item analysis was performed for the SC test to objectify difficulty and selectivity.

**Results:**

Statistically significant, the mARble group (6/10) showed greater knowledge gain than the control group (4/10) (Wilcoxon *z*=2.232, *P*=.03). The item analysis of the SC test showed a difficulty of *P*=0.768 (s=0.09) and a selectivity of RPB=0.2. For mARble, fatigue (*z*=2.214, *P*=.03) and numbness (*z*=2.07, *P*=.04) decreased with statistical significance when comparing pre- and post-tests. Vigor rose slightly, while irritability did not increase significantly. Changes in the control group were insignificant. Regarding hedonic quality (identification, stimulation, attractiveness), there were significant differences between mARble (mean 1.179, CI −0.440 to 0.440) and the book chapter (mean −0.982, CI −0.959 to 0.959); the pragmatic quality mean only differed slightly.

**Conclusions:**

The mARble group performed considerably better regarding learning efficiency; there are hints for activating components of the mAR concept that may serve to fascinate the participants and possibly boost interest in the topic for the remainder of the class. While the small sample size reduces our study’s conclusiveness, its design seems appropriate for determining the effects of interactive eLearning material with respect to emotions, learning efficiency, and hedonic and pragmatic qualities using a larger group.

**Trial Registration:**

German Clinical Trial Register (DRKS), DRKS-ID: DRKS00004685; https://drks-neu.uniklinik-freiburg.de/drks_web/navigate.do?navigationId=trial.HTML&TRIAL_ID=DRKS00004685.

##  Introduction

Mobile Augmented Reality (AR) offers valuable learning opportunities and may have the potential to significantly improve the learning environment and the attractiveness of the learning process. Mobile AR blended learning environments offer a new level of experience for learners, especially in areas such as forensic medicine where ethical constraints may have to be placed on learning specific subjects in real-life scenarios. For nonmedical education, a number of studies have shown beneficial effects for AR-supported study modules. Many of these make use of AR in a mobile setting [1,2]. If used appropriately, this allows users to “immerse” themselves in the subject at hand [3] and to become involved in their own learning process.

Although there are a number of projects that integrate mobile AR for basic science education, for example, for middle-school or high-school students—and some of these also touch on subjects related to medicine [4]—projects employing such concepts for basic medical education are still rare. Regarding medicine in general, AR-based applications have so far been put to use mostly for supporting diagnostic or therapeutic purposes [5,6]. Other projects provide more or less complex simulations, such as for surgical training [7]. Although these approaches generally use Augmented Reality for complex scenarios, they all have in common that the technology is used in a stationary way that—even when used for educational purposes—keeps the users emotionally detached from the subject at hand. Often, they only serve to teach physicians about the use of specific tools, such as laparoscopic tools [7], or diagnostic methods, where a certain distance to the patient would be kept even in real-life scenarios. Although such projects certainly enhance learning by giving users experiences they would otherwise not be able to have, the aforementioned stationary AR-based diagnostic and training applications also usually do not allow full immersion of the users into the learning experience. They do not make them an integral part of the learning experience, for example, by projecting the learning content on the learner’s body and thus potentially evoking emotional responses in them that might have an additional influence on the learning process.

The current paper describes a methodological approach and study design that can be used for the purpose of measuring basic cognitive and emotional factors that must be dealt with when integrating AR-based mobile applications into medical teaching. To allow experimental testing of the aforementioned approach and study design, a mobile AR-based prototype app (mARble) was developed that can serve to provide medical students and their educators with a versatile mobile learning environment, making it possible to simulate situations that are either ethically problematic or only rarely encountered in real life [8-11]. This prototype included content for forensic medicine. Education in this field often suffers from specific cases either being unavailable or unusable due to ethical restrictions, since—especially when dealing with survivors of a crime—additional traumatization must be strictly avoided.

In the context of this mobile learning environment, the mobile device serves to meet two basic demands for almost realistic wound pattern simulations. First, it is a portable and highly capable multimedia device, making it an ideal choice for using the technology in various learning situations. Second, through its highly advanced features, it even allows for augmented reality in these learning situations. Thus, it becomes possible to provide new, more realistic elements for the learning setting, such as the projection of wound patterns on the skin of the students, and to possibly provide a new learning experience. Using such an approach, the learners themselves become objects in their own learning process. Thus, they may more easily identify themselves with their role as a patient or an assault victim.

So far, little is known about the impact of mobile AR applications on the learner during the learning process. It is still unclear which emotions and cognitive effects are provoked in the recipient due to a higher level of realism combined with a very personal experience in a simulated setting. According to Edelmann [12], emotions may have an influence on various aspects of learning. When an individual processes information, facts are attributed with “subjective significance” based on the triggered emotions and thus become a part of that individual’s interpretative system. Depending on the perceived success or failure of the learning process, for example, determined by exams, this can also have an effect on an individual’s subjective well-being [13]. In general, emotions that are evoked while learning are not only important when considering single individuals. They also have a big influence on the communication processes within groups of learners as well as with their teachers and are thus one of the key factors for overall learning success.

When taking a closer look at the significance of emotions on the learning process, a number of important questions arise: How can emotions be classified? How can their effects and benefits for the learning process be reliably quantified? In literature, there is currently no uniform scheme that sufficiently covers all of these aspects. This is additionally complicated by the fact that the impact of emotions also depends on the sociocultural context [14]. Another problem is that it is hard to differentiate emotional aspects from related psychological concepts such as “motivation” [13]. The influence of certain emotions on the success of specific learning methods, for example, if someone is in favor of authoritative or more liberal teaching methods, may also depend on an individual’s ideological perspective [13]. When trying to describe emotions, subjective assessments must also be taken under consideration since terms such as “disgust”, “modesty”, “fear”, or “insecurity” may not always describe the exact same emotions for different individuals. It is also difficult for people to quantify their emotions exactly since emotions are regularly perceived on an instinctive, subjective, and nonverbal level.

For the purpose of the current paper, three core dimensions were identified for the evaluation of our AR-based learning environment, specifically to be able to confirm our expectations that emotional involvement during the learning process as well as learning efficiency for students learning with mobile augmented reality rise compared to those using only textbooks.

The first dimension was defined as learning effectiveness, which quantifies the influence of a learning method on the acquisition of knowledge. It was of special interest to investigate whether an improvement in knowledge is possible by means of training based on a specific learning method. The second dimension deals with the learning experience itself. This includes the usability of the provided material (practicability), the user’s identification with the learning method, the stimulation it provides, as well as its attractiveness for students and educators. The third dimension that was identified as having an influence on learning success, as indicated above, is emotion. There may be a change in the emotional status of learners after using a certain learning method. This could speak to an additional emotional involvement that may be due to the chosen learning method, for example, additional realism when using modern tools and applications that integrate augmented reality. The students become their own learning subjects, which offers a chance for experiencing an additional layer of learning: potential personal involvement.

##  Methods

### Participants

Ten third-year medical students (6 male, 4 female, mean age: 23.7 years, standard deviation: 2 years) were included in the prestudy after giving their informed consent to participate in the trial. The students had not previously participated in any regular courses dealing with the learning topic presented during the trial. Since all participants had already completed the mandatory curriculum of medical informatics, where, aside from theoretical knowledge, they were also introduced to practical aspects of using computers, it was assumed that all of them had attained at least a basic level of computer literacy.

The study was approved by the Institutional Review Board of Hannover Medical School, (ID: 1653-2012).

**Figure 1:**
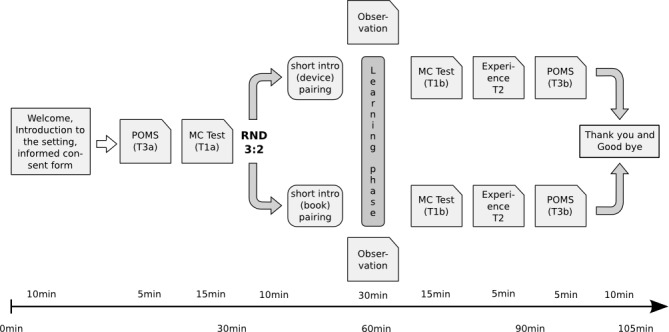
Timeline and applied tests. In the text, individual blocks are referenced via labels (T1a/b, T2, and T3a/b).

As shown in Figure 1, to measure the emotional state of the students before the training session, all students were asked to fill out the German variation of the “Profile of Mood States” (POMS) questionnaire within a period of 5 minutes (T3a). To establish a baseline with respect to a priori knowledge of the learning topic, a 10-question standard multiple choice test about “gunshot wounds” (T1a) was given to the students, which they were asked to complete within 15 minutes. After the initial testing, the students were randomly assigned to two subgroups, named group A (6 students) and group B (4 students). Group B participated in the conservative learning situation, finding themselves in a quiet room and reading a 10-page excerpt from a standard textbook in forensic medicine [15] about “gunshot wounds”. The students were instructed to read and learn about the topic using the textbook material for a learning period of 30 minutes. While learning, they were allowed to use additional supplies such as pencils, pens, and paper to take notes and highlighters to work in the provided copies of the text material. Also, the students were free to discuss the learning topics and were specifically instructed to interact freely with other participants in their group. After 30 minutes, the students were again asked to complete the previous standard multiple choice tests comprising 10 questions (T1b). During the tests, the participants were not allowed to refer to the textbook material or their notes; they were given 15 minutes to complete the test (10 questions, 90 seconds for each answer). Afterwards, the students were asked to provide information about their learning experience (T2, 10 minutes). The POMS (T3b) questionnaire was administered to determine their emotional state after the training session. During the trial, a member of the study personnel was placed in the same room for direct observation (T4) and also to provide feedback to the students if necessary. At the end, the study participants were thanked and invited to come back on another day if they wished to try out mARble.

Group A joined the interventional arm of the trial. The group was divided into 3 pairs and each subgroup received an iPhone 4, on which the app “mARble Forensics” had already been installed, and a set of 3 paper markers. After a short greeting and introduction to the application, working with the provided markers and the learning task, the 3 pairs of students were directed into the corners of another quiet room, away from group B. The task was to learn about “gunshot wounds” using the provided iPhone and the preinstalled mARble application. The information in the mARble application contained all information relevant for later solving the multiple choice test. After 30 minutes, the students of group A had to solve the same tests as those in group B (T1b), including the multiple choice test, a questionnaire about their learning experience (T2), and the POMS questionnaire (T3b) to determine their emotional state. Just as for group B, during the trial, a member of the study personnel stayed with the students of group A for providing feedback and for direct observation (T4). After completion of all tests, participants were thanked and dismissed. The complete timeline of the individual test elements for both groups is shown in Figure 1.

###  Learning Material Provided

####  The Application

mARble is an iOS application that was developed at the Peter L. Reichertz Institute for Medical Informatics (PLRI) at the Hannover Medical School. Using AR, virtual information can be linked to objects in the real environment, thus providing an additional layer of information to the users [10]. Code and content for mARble are kept separately. Information can easily be edited or added based on an XML-based file format. The content for the forensic module of mARble used during the course of our study was derived from and corresponded to the textbook-based learning material that was provided to the group of learners belonging to the “conventional” learning group.

**Figure 2:**
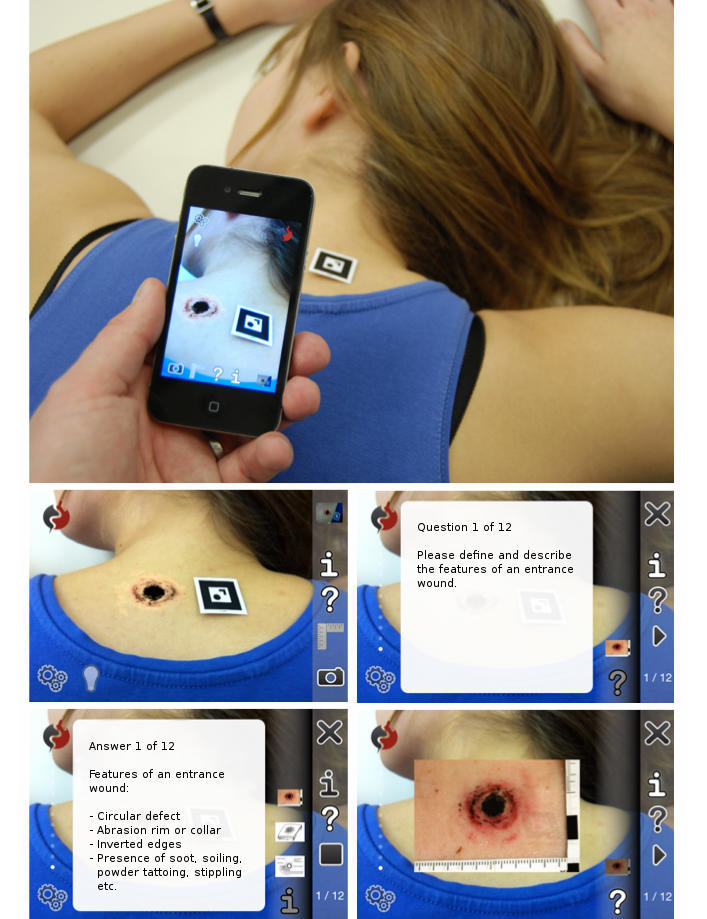
The mobile Augmented Reality blended learning environment with the module “Forensic Medicine”: AR simulation of a gunshot wound and connected multimedia content.

Based on this module, mARble was able to detect and interpret predefined markers representing various pathologies commonly found in forensic medicine. Each marker corresponded to a wound pattern that the students were expected to explore. When placing a marker on the student’s body, for example, on the neck, the image acquired by the iPhone’s camera was automatically overlaid with the corresponding wound pattern, such as the entrance wound of a bullet, as seen in Figure 2. Through the virtual flashcard system included in mARble, it was also possible to view textual and multimedia background information (ie, images, drawings, video, and audio) linked to the current marker and work with the provided questions and tasks (Figure 2). Through the described approach, learners were able to construct various fictive cases by combining markers for the desired set of findings. The learning process could be documented by adding snapshots of the augmented image to a personal image gallery. Previously taken snapshots and findings could be used for review, for discussions with fellow students, or presentation purposes. When reviewing an image, it was also possible to trigger the corresponding background information as well as associated questions and tasks. Using their iPhone, students were able to examine the provided wound patterns either on themselves or on their partner; thus, they could easily immerse themselves in the learning topic.

####  The Conventional Learning Material

We chose the 10-page chapter “gunshot wounds” of a popular short compendium of forensic medicine in Germany [15] as learning material for group B. The textbook is very well-equipped with color and black/white pictures, schemes, and tables, as well as small repetitive summaries in colored boxes. Roughly 50% of the material consists of images and drawings.

###  Overall Learning Experience: Evaluation Tools

####  Learning Success: Multiple Choice Test (T1a, T1b)

A paper-based test consisting of 10 questions with single choice answers was used to measure the learning effectiveness. The questions and related answers were collected from a pool of material compiled by a member of the staff of the forensic medicine department. Before the trial, two members of the staff evaluated the multiple choice questionnaire with respect to comprehensibility, solvability, and time consumption. Beforehand, both the textbook extracts as well as the content provided in mARble were reviewed to determine whether the content necessary for answering all questions was sufficiently covered. Also, an item analysis before and after the learning period was conducted to take into account test difficulty (p), item discrimination (RPB), and item selectivity.

#### Statistical Analysis

To determine learning effectiveness (T1a and T1b), descriptive statistics were calculated, including the mean, standard deviation (SD), and mean for the score in total and the subgroups, before and after the intervention. In a noninferiority design (unpaired rank sum, Mann-Whitney U, 2-sided, Cronbach alpha=.05), the scores reached by learning with mARble were tested against the scores achieved when using the classical learning material. The calculation of T1a and T1b was based on the sum of the item values. All questionnaires were included. Those with one missing item per scale were corrected with the scale mean.

####  Learning Experience (T2): AttrakDiff2

Over the past few years, “user experience” has become an important factor for the acceptance of all technical innovations. Nevertheless, only vague definitions exist and there are many unanswered questions concerning the factors contributing to a good user experience [16]. Hassenzahl et al [17-19] designed a model that classifies the attributes necessary for describing products according to their pragmatic or hedonic quality and can thus be employed to describe the subjective attractiveness. This experience design concept was integrated into the test design. Following Hassenzahl’s theoretical work model, the pragmatic and hedonic qualities of an application influence a user’s subjective perception of attractiveness, resulting in respective behavioral and emotional responses. In this context, hedonic quality describes the emotional impact of a product or system, while measuring pragmatic quality offers insights into its usability or usefulness [20].

AttrakDiff2 [19] was developed as a tool by Hassenzahl’s research group to be able to quantify these qualities. The tool uses 4x7 anchor scales, in total 28 questions. The anchors are presented in the form of semantic differentials and a 7-point Likert scale is employed for rating the intensity of the items. The poles of each item are opposite adjectives (eg, “confusing-clear”, “unusual-ordinary”, “good-bad”). Each of the mean values of an item group creates a scale value for pragmatic quality (PQ), hedonic stimulation (HQ-S), hedonic identification (HQ-I), and attractiveness (ATT).

Attributes in the PQ group describe how easy the user finds it to work with the provided program or environment. Pairs of words belonging in this group are for example “technical vs human”, “complicated vs simple”, or “impractical vs practical”.

Attributes belonging to HQ-S describe factors that encourage the personal growth of users and provide stimulation to give them the opportunity to enhance their knowledge and development. Stimulating factors can be delivered in many different ways, such as by presenting things in a novel way or by providing a new interaction style. Anchors for hedonic stimulation include attributes such as “professional vs unprofessional”, “stylish vs tacky”, or “isolating vs connective”.

The attributes falling into the HQ-I category make it possible to identify the social impact that using a product can have for users, including the “messages” that are communicated by using the evaluated product [20]. Anchors belonging in this group are, for example, “ordinary vs novel”, “conservative vs innovative”, or “undemanding vs challenging”.

Last, the attributes of the ATT group depict the overall experience a product has to offer to its users, that is, its attractiveness. Contributing attributes are, for example, “pleasant vs unpleasant”, “ugly vs attractive”, or “appealing vs unappealing”.

#### Statistical Methods

A Mann-Whitney U test for independent random sampling (Cronbach alpha=.05) was conducted to discriminate a possibly significant difference within the categories. Overall scores for the individual 28 attributes as well as aggregated values for each category were obtained by calculating the average values of the ratings provided by the users. To better visualize the relationship between pragmatic and hedonic qualities, the values calculated for PQ are shown on one axis and the values for HQ-I as well as HQ-S on the other axis. Combined with the confidence interval (CI) for each value, the values obtained for the ratings allow a clear differentiation between students using text-based learning or mARble.

####  Emotional Involvement (T3a+T3b): POMS Questionnaire, German Version

To measure the emotional state and possible psychological distress before (T3a) and after (T3b) the learning phase, we asked all students to answer the German variation of the POMS questionnaire by McNair et al [21]. This version, modified by Biehl, Dangel, and Reiser [22], consists of 35 items (adjectives) that can be divided into 4 groups describing mood disturbances, including fatigue-inertia (14 items), vigor-activity (7 items), tension-anxiety (7 items), and depression-dejection (7 items). Participants rated each item on a 7-point rating scale according to the experienced intensity (eg, “not at all”, “very little”, “a little”, “somewhat”, “fairly”, “strongly”, “very strongly”) of the corresponding mood disturbance. The triggering question was formulated as: “How do you rate your current emotional state?”

The study participants were asked to finish the survey in approximately 5 minutes. Internal consistency estimates range between Cronbach alpha=.89 and Cronbach alpha=.95 [23]. We decided to use this instrument due to its known validity and its broad usage in medical [24-26] and psychological [27] disciplines.

####  Direct Observation of the Participants (T4)

To be able to extensively evaluate the learning situation, observations were included in the study design. A nonparticipant observation was chosen for data collection. During the trial, the behavior of participants from both groups was observed by trained personnel. The observers, all having at least 5 years of experience teaching at a university level, were required to focus on a priori defined criteria of learning behavior as the primary basis for organizing and reporting results. Notes of additional observations of any kind were allowed and were used for further qualitative analysis. The development of observation criteria referred to statements of Schulmeister [28] on learning psychology-based factors of virtual teaching and learning, where the importance of social presence in learning settings is emphasized.

This well-known classification provided the basis for observing both groups in order to examine possible differences in participants’ learning behavior. According to this classification, the following observation criteria have been selected: (1) student’s communication and interactivity with peers, (2) student’s focus on or distraction from the learning material, and (3) the way the student dealt with the learning object (learning material).

##  Results

###  Learning Success: Multiple Choice Test (T1a, T1b)

**Figure 3:**
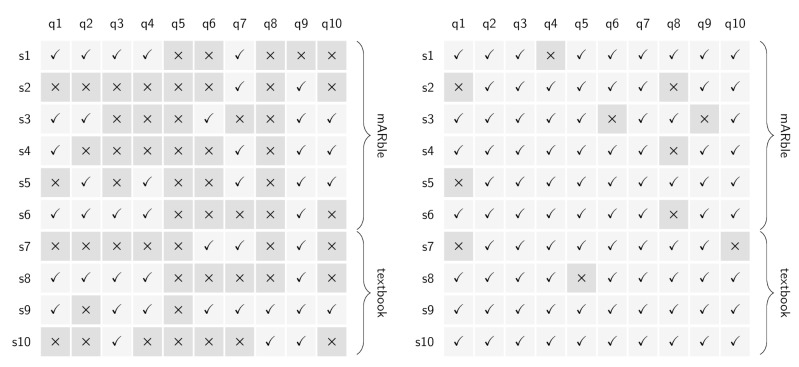
Number of incorrectly and correctly answered questions before (left) and after (right) the learning period.

Comparing the results of the multiple choice tests before and after the learning period, on average, all participants showed an improvement regarding correct answers (Figure 3). Stratified for the learning method, the improvement was higher in the mARble group with 4.7 questions (SD 2.9) compared to the control group showing an improvement of 3 questions, but also with smaller variability (SD 1.5). The difference in improvement within the mARble group was statistically significant (Wilcoxon, *z*=2.232, *P*=.03). The multiple choice test difficulty was calculated with *P*=0.768 (SD 0.09) with an item discrimination of RPB=0.2.

###  Learning Experience (T2): AttrakDiff2

**Figure 4:**
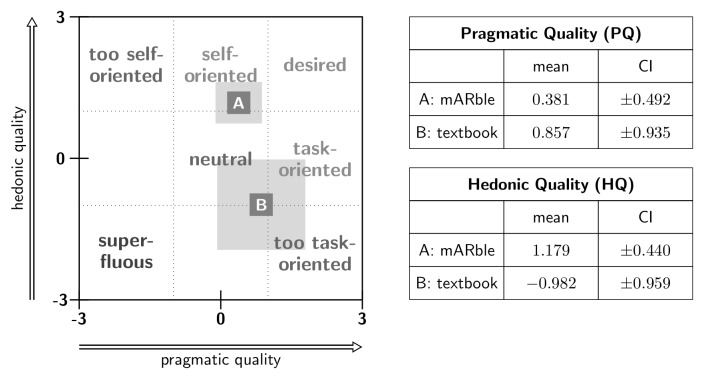
Portfolio with average values of the dimensions PQ and HQ and the respective confidence rectangles of A (mARble) and B (textbook) on left, modified following Hassenzahl et al; corresponding values on right.

**Figure 5:**
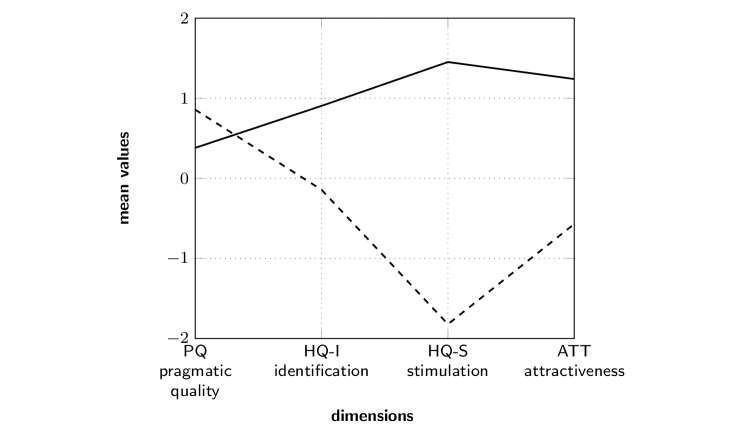
Average values for pragmatic quality (PQ), hedonic quality – identification (HQ-I), hedonic quality – stimulation (HQ-S), and attractiveness (ATT), based on evaluation of the AttrakDiff2 questionnaire (solid line: mARble group (6/10); dashed line: textbook group (4/10)).

**Figure 6:**
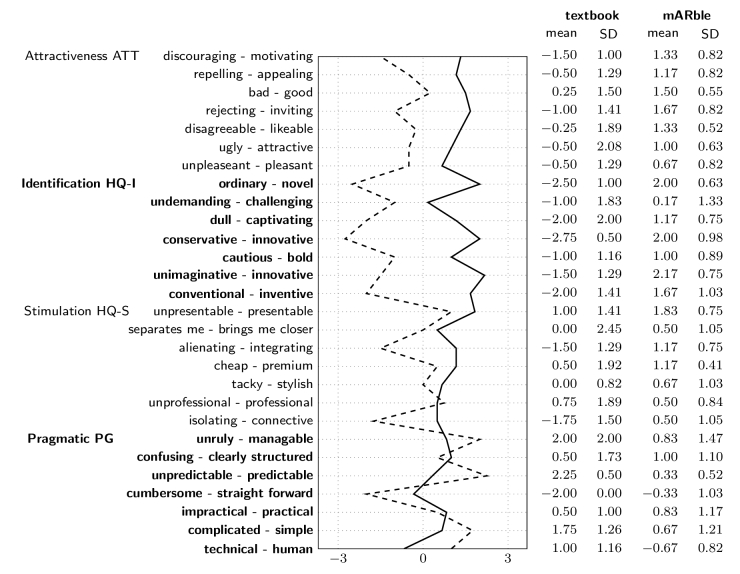
Description of word-pairs and calculated values: comparison between mARble (solid line) and the textbook material (dashed line).

Statistical analysis revealed significant differences between the mARble and the textbook groups (Table 1) for the hedonic qualities, HQ-S “stimulation” (Mann-Whitney U, *z*=6.506, *P*<.001), HQ-I “identification” (Mann-Whitney U, *z*=2.825, *P*=.005), and ATT “attractiveness” (Mann-Whitney U, *z*=5.179, *P*<.001). mARble obtained more positive ratings. The confidence interval (CI) for the hedonic quality of the mARble group was smaller than for the textbook group (Figure 4), since mARble’s users were more consistent in their evaluation; therefore, mARble’s ratings were applied with greater certainty. When comparing the values for pragmatic quality, the textbook group performed better than the mARble group, although this difference is not statistically significant (Mann-Whitney U, *z*=−1.616, *P*=.11). However, for the identity aspect of hedonic quality, the mARble group performed significantly better than the textbook group (Mann-Whitney U, *z*=2.825 *P*=.005). Furthermore, considering the hedonic quality “stimulation”, participants in the mARble group performed much better than the textbook group (Mann-Whitney U, *z*=6.506, *P*<.001). This resulted in a difference in participants’ ratings of attractiveness (Mann-Whitney U, *z*=5.179, *P*<.001), with mARble again receiving better ratings (Figures 4 and 5). Figure 6 describes the profile of the mean values and standard deviations for the word pairs stratified for the learning methods.

**Table 1:** Aggregated values calculated for the 4 qualities covered by AttrakDiff2: pragmatic quality (PQ), identification (HQ-I), stimulation (HQ-S), and attractiveness (ATT).

Group	PQ, mean (SD)	HQ-I, mean (SD)	HQ-S, mean (SD)	ATT, mean (SD)
A: mARble (n=6)	0.381 (1.168)	0.9048 (0.932)	1.452 (1.087)	1.24 (0.726)
B: textbook (n=4)	0.857 (1.758)	−0.143 (1.780)	−1.821 (1.39)	−0.57 (1.451)

###  Emotional Involvement (T3a+T3b): POMS Questionnaire, German Version

**Figure 7:**
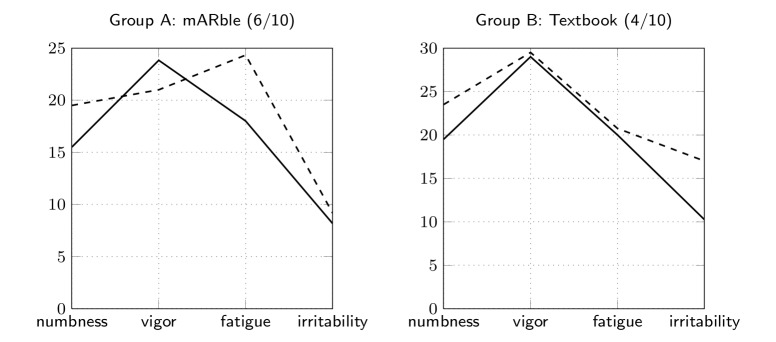
Learning affection: pre-test (dashed line) and post-test (solid line) comparison of the aggregated values for numbness, vigor, fatigue, and irritability for both groups.

In our study, POMS was used to measure a change in the emotional state change before and after learning. The answers according to the dimensions numbness, fatigue, vigor, and irritability are shown in Table 2 and visualized in Figure 7 and were aggregated from the item values recorded for both groups of participants. A comparison of pre- and post-test values (Figure 7) showed a statistically significant decrease of fatigue (*z*=2.214, *P*=.03) and numbness (*z*=2.07, *P*=.04) for the mARble group; vigor increased slightly. Irritability did not change significantly (*z*=1.166, *P*=.24). The control group did not show significant changes on any of the variables.

###  Direct Observation of the Participants (T4)

####  Group A: mARble

Nonparticipant observations of the two groups showed a highly heterogeneous pattern of results. Group A (alternative learning method mARble) comprised 6 participants (P1-6) that were assigned to one of three subgroups (SG1 to SG3). In the beginning of the learning phase, all students explored the functionality of the mARble app on the iPhones. They used the marker and tried to get the picture of the linked object on their iPhone display. There was one group consisting of male participants, one with female participants, and one mixed group. Both same-sex groups showed a high level of interaction, placing the markers on their bodies, discussing the content, and taking notes. Although the mixed group also interactively used and discussed the content, they refrained from placing the markers on their skin, instead simply placing them on the table.

####  Group B: Textbook

Group B (conservative learning method) consisted of 4 participants (P1-P4) being paired and assigned to two subgroups (SG1: male, male; SG2: female, male). All participants were instructed to learn in their usual manner, but also asked to discuss the text material with their learning partner and with the whole group if they desired. Still, there were almost no dialogues with either the whole group or between the members of SG1 or SG2. From the beginning of the learning phase, all participants worked in a focused and concentrated manner, and read the text quietly. There were only very short interruptions: two participants briefly talked to each other and there was one distraction due to disruptive environmental influences. Only near the end of the learning phase was there some exchange between the participants. This did not exclusively relate to the learning content but also encompassed private matters. Further kinds of interactivity (other than communication) did not take place. The way students had worked with the text could be tracked by looking at what they had highlighted or underlined.

**Table 2:** Aggregated values for numbness, vigor, fatigue, and irritability.

Group	Phase	Dimensions			
		Numbness, mean (SD)	Vigor, mean (SD)	Fatigue, mean (SD)	Irritability, mean(SD)
**A: mARble (n=6)**					
	pre	19.5 (4.637)	21.0 (4.561)	24.33 (3.204	9.17 (2.041)
	post	15.5 (3.209)	23.83 (9.326)	18.0 (4.147)	8.17 (1.472)
**B: textbook (n=4)**					
	pre	23.5 (2.887)	29.5 (9.037)	20.75 (9.570)	17.0 (5.944)
	post	19.5 (4.041)	29.0 (7.616)	20.0 (7.528)	10.25 (3.594)

##  Discussion

### Principal Findings

The use of mobile devices, especially when augmented reality comes into the picture, can considerably change the learning experience as well as shift it to an entirely new level [3,29], thereby providing learning experiences that are simply not possible in a conventional learning setting.

For the study, a mobile AR learning environment was developed for almost realistic wound pattern simulations in medical settings, where learners become emotionally involved in their learning process. For us, it was important to determine whether the use of AR-based solutions might trigger negative emotions or irritations in learners: when learning with mARble, by placing the markers representing specific findings on their bodies and viewing the findings on their own skin, they become emotionally involved and a part of the learning process. For the current study, we carefully investigated the emotional and cognitive impact mobile AR applications might have on the learner and the learning process in contrast to a control group of subjects that learned in a conventional medical learning environment. Since emotional reactions are often domain-specific [30], the control group served to make sure that the measured effects were not caused by the content but by the learning medium.

Although our study showed that both learning environments induced significant cognitive improvement with respect to an increased knowledge, in direct comparison, the mARble group performed significantly better.

Regarding pragmatic quality, both methods were given average ratings. While mARble’s user interface was interpreted as fairly self-oriented, the textbook was rated as rather task-oriented. Nevertheless, mARble is much more attractive to its users. The ratings also point to a significant stimulation offered by mARble. Solely in terms of pragmatic quality, the textbook material was located in the above-average region. It meets ordinary standards with regard to hedonic quality–identity. For hedonic quality–stimulation, the textbook is located in the below-average region. It does not have a stimulating effect on users. Insufficient stimulation results in a lack of motivation when using the product. Should products of similar pragmatic quality be available, users would gladly change products. The attractiveness value is located in the average region. The overall impression of the product is moderately attractive.

In comparison to the textbook material, mARble obtained better ratings with respect to vigor; its users were less fatigued after using mARble. There was no indication of irritating properties for either of the two learning methods. Similarly, during observation, the participants showed no signs of emotional irritation. Nonetheless, the different behavior of the participants in the two groups suggests that learning with mARble and using markers on the body might provoke emotions such as shame or shyness. The participants in the gender-mixed groups did not use the markers directly on their skin but only on the table’s surface. However, these aspects require further investigation.

### Limitations

It might be argued whether the observed results were due to the medium, textbook vs mARble, or rather due to the chosen approach, individual learning vs social interaction. Both factors are probably closely interrelated. For example, for the control group learning with the textbook material, discussion and interaction between the participants was not prohibited in any way; rather, students were specifically asked to interact with other members of their learning group if they desired. Although they sat close to each other, they chose individual learning instead of social interaction. However, due to the limited number of participants in this prestudy, we refrained from specifically using separate groups for testing both textbook and mARble in individual learning sessions as well as in an interactive way, and instead let the participants choose what suited them best.

Nevertheless, the effects of both learning approaches cannot be completely separated from the effects of the chosen learning material. Even if students learning with textbooks were to choose social interaction during their learning phase, they would still need time periods to read the material. On the other hand, with AR-based approaches, they can collaboratively use the presented material by listening to the content, looking at overlaid images and additional material right away, which encourages social interaction. Still, the possible bias of the results we achieved by direct comparison between the textbook-based learning, which is assumed to be an individual learning method when the students are not explicitly ordered to collaborate vs the AR-based learning, where we expected a more collaborative learning process by deploying one device to each pair of students, remains a limitation of the presented study. An upcoming study should consider this and take the effects of social interaction or missing interaction during the learning process into account, for example by using individual as well interactive setups for both textbook-based and AR-based learning and comparing the results not only between the learning methods themselves but also between collaborative as well as individual learning settings for both methods.

Additionally, the multiple choice questions used for rating the students’ performance should be selected more carefully. When looking at the results of the item analysis of the multiple choice questions, it became clear that the item difficulty and item discrimination of the questions used during the presented study still had room for improvement. This could, for example, be alleviated by selecting items where the values for these criteria are already known, such as by choosing questions from previously conducted official exams rather than self-developed items.

### Conclusions

Although limited by the small sample size, and the limited amount of time and content available, the chosen evaluation setup allowed for certain conclusions regarding the desired factors; a study using a larger group of participants, based on our current study’s design, may provide more conclusive results regarding various aspects of interactive mobile learning tools such as mARble in comparison to conventional learning material.

## Abbreviations

AR: Augmented Reality
mAR: mobile Augmented Reality
mARble: mobile Augmented Reality blended learning environment
PLRI: Peter L Reichertz Institute for Medical Informatics
POMS: Profile of Mood States questionnaire
SC: single choice
